# Solid pancreatic masses in children: A review of current evidence and clinical challenges

**DOI:** 10.3389/fped.2022.966943

**Published:** 2022-11-25

**Authors:** Kelli N. Patterson, Andrew T. Trout, Archana Shenoy, Maisam Abu-El-Haija, Jaimie D. Nathan

**Affiliations:** ^1^ Center for Surgical Outcomes Research, Abigail Wexner Research Institute, Nationwide Children's Hospital, Columbus, OH, United States; ^2^ Department of Radiology, Cincinnati Children's Hospital Medical Center, Departments of Radiology and Pediatrics, University of Cincinnati College of Medicine, Cincinnati, OH, United States; ^3^ Department of Pathology and Laboratory Medicine, Nationwide Children's Hospital, Department of Pathology, The Ohio State University College of Medicine, Columbus, OH, United States; ^4^ Division of Gastroenterology, Hepatology and Nutrition, Cincinnati Children's Hospital Medical Center, Department of Pediatrics, University of Cincinnati College of Medicine, Cincinnati, OH, United States; ^5^ Department of Abdominal Transplant and Hepatopancreatobiliary Surgery, Nationwide Children's Hospital, Department of Surgery, The Ohio State University College of Medicine, Columbus, OH, United States

**Keywords:** pancreas, pediatrics, child, neoplasms, general surgery, surgical oncology

## Abstract

Pancreatic tumors in children are infrequently encountered in clinical practice. Their non-specific clinical presentation and overlapping imaging characteristics often make an accurate preoperative diagnosis difficult. Tumors are categorized as epithelial or non-epithelial, with epithelial tumors further classified as tumors of the exocrine or endocrine pancreas. Although both are tumors of the exocrine pancreas, solid pseudopapillary neoplasm is the most prevalent solid pancreatic tumor in children, while pancreatoblastoma is the most common malignant tumor. Insulinoma is the most common pediatric pancreatic tumor of the endocrine pancreas. Malignant tumors require a complete, often radical, surgical resection. However, pancreatic parenchyma-sparing surgical procedures are utilized for benign tumors and low-grade malignancy to preserve gland function. This review will discuss the epidemiology, pathophysiology, clinical and diagnostic characteristics, and management options associated with both common and rare solid pancreatic masses in children. We will also discuss current challenges encountered in their evaluation and treatment.

## Introduction

Pancreatic tumors are rare in children. An age-adjusted annual incidence of 0.19 cases per million pediatric population has been estimated in North America (1). Research and evidence-based protocols are, therefore, limited. Often, pediatric pancreatic tumors are difficult to differentiate due to their overlap in non-specific clinical presentation and diagnostic imaging characteristics. In general, pancreatic tumors most commonly diagnosed in children tend to be well-circumscribed lesions without invasiveness. Additionally, in contrast to adults, children and adolescents with malignant pancreatic tumors tend to have disease which is amenable to surgical resection and longer expected survival.

According to the 2019 World Health Organization (WHO) definition, pancreatic tumors can be divided into three categories including benign epithelial tumors, malignant epithelial tumors, and neuroendocrine tumors, with an additional group of rare non-epithelial tumors (2). Epithelial tumors may also be categorized as tumors of the exocrine or endocrine pancreas, with exocrine tumors being of acinar, ductal, or unknown cell origin (3) (Table 1). This review discusses the epidemiology, pathophysiology, clinical and diagnostic characteristics, and management options associated with solid pancreatic masses in the pediatric population. We also discuss the challenge of differentiating autoimmune pancreatitis from pancreatic malignancy, as well as the approach to parenchyma preservation during resection of pancreatic masses.

**Table 1:** Solid pancreatic tumors diagnosed in children with associated clinical characteristics.

Tumor type	Cell origin; category	Mean age of children affected (years)	Size at diagnosis (cm)	Overall Prognosis
**Epithelial tumors**				
Acinar cell carcinoma	Acinar cell; exocrine	Rare	10–11	5-year survival rates of up to 50% for localized disease with R0 resection
Pancreatic ductal adenocarcinoma	Ductal cell; exocrine	Rare; 1% occur in patients under 20	2–3	15-year survival rate of 23% in children
Pancreatic neuroendocrine tumor *Insulinoma most common	Endocrine cell (*beta cell); endocrine	*4.9% found in children 10–19; 0.9% found in children 0–9	*∼2 cm	15-year survival rates of 50% across pancreatic neuroendocrine tumors overall
Pancreatoblastoma	Acinar cell; exocrine	4–5	5–20	5-year overall survival rates >70% with R0 resection and no metastatic disease
Solid pseudopapillary neoplasm	Unknown; exocrine	13–14	5–7 (range <0.5–20)	10-year survival rates >95% with R0 resection even in presence of metastatic disease
**Non-epithelial tumors**				
Dermoid cyst		Rare	8–12	Benign; good prognosis
Inflammatory myofibroblastic tumor		Rare (range 6 months – 15 years)	1.5–15	Good; low-grade malignancy with no recurrences after complete surgical resection
Kaposiform Hemangioendothelioma		Infancy and early childhood	>8 with KMP	Mortality rate 12%–24%; often with KMP
Lymphatic malformation		Rare	3–20	Benign; good prognosis
Pancreatic Ewing sarcoma		18.2 (range 2–37)	3.2–22	Overall (pancreatic and extra-pancreatic) 5-year survival of 55%–65% with localized disease and multimodal approach
Pancreatic primary lymphoma		10.3 (range 3–16)	5–6	Good; 15 patients - all alive after 56 months follow-up with IC and/or surgery

KMP, Kasabach-Merritt phenomenon; IC: immunochemotherapy.

*Statistics for insulinoma.

## Pancreatoblastoma

### Epidemiology

The most common malignant pancreatic neoplasm in children is pancreatoblastoma (PBL), which accounts for 25% of solid pancreatic masses in the first 10 years of life (4). Nonetheless, PBLs are rare with a recent systematic review identifying 81 pediatric cases since 1980 (5). PBLs are typically diagnosed in the first decade of life, with the median age being 4–5 years old (3, 6–8), although there are several cases reported in older children and adults (9–12). Boys are more affected than girls.(3, 8, 13, 14). PBL has been shown to have an association with Beckwith-Wiedemann syndrome, with a prevalence up to 50% in patients diagnosed (15, 16). One case has also been reported with concurrent familial adenomatous polyposis (FAP) (15, 17). PBL is an embryonal tumor originating from pluripotent pancreatic stem cells during the gestational development of foregut structures (7, 18–20). Its molecular pathogenesis has similarities to that of hepatoblastoma, with abnormalities in the adenomatous polyposis coli (APC)/beta-catenin pathway and chromosome 11p. This may explain its reported association with FAP and suggests that PBL could be an extracolonic manifestation of the disease (15–17).

### Gross pathology and histology

**Figure 1:**
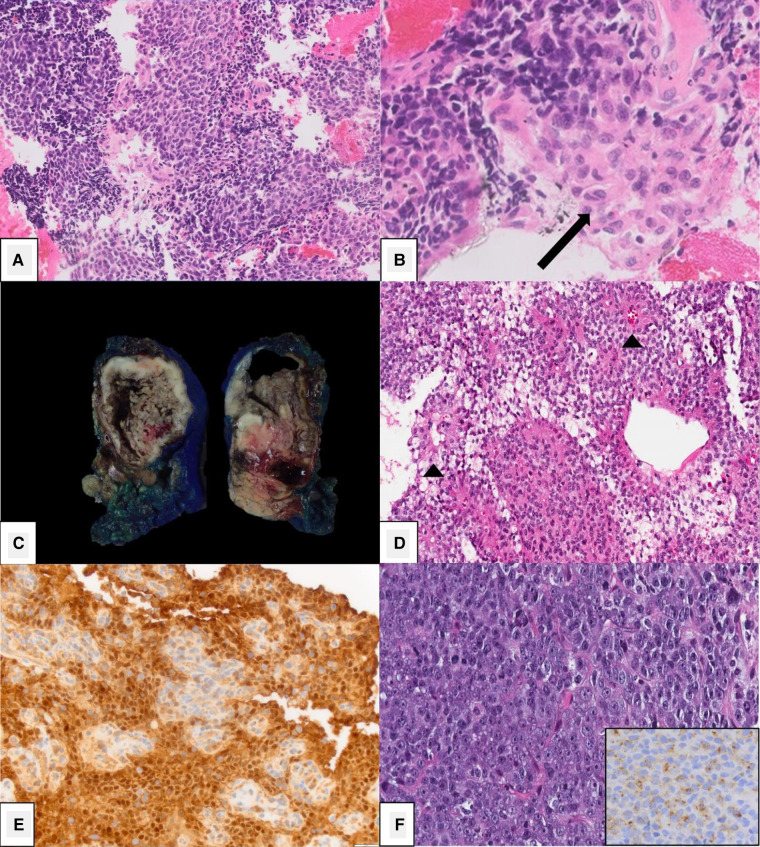
Histopathology. (**A-B**) Pancreatoblastoma characterized by rounded cells with eosinophilic cytoplasm and diagnostic squamoid corpuscles (arrow, B) [Picture courtesy: Kathleen Byrnes, MD, Washington University in St. Louis, MO]. (**C-E**) Solid pseudopapillary neoplasm demonstrating a solid and cystic cut-surface (**C**), pseudopapillary structures with central vascular cores (**D**) and nuclear beta-catenin positivity by immunohistochemistry (**E**). (**F**) Acinar cell carcinoma demonstrating a high-grade malignancy composed of round to oval cells with moderate granular amphophilic cytoplasm, prominent nucleoli and positive trypsin immunohistochemistry.

Tumors are generally located in the head or body of the pancreas (5, 15). They are soft, circumscribed, and large (5 cm–20 cm in size). They are also frequently either partially or fully encapsulated. On cut surface, lesions are tan, lobulated, and separated by thick fibrous bands with associated hemorrhage and necrosis. Cystic changes may be appreciated, especially in patients with Beckwith-Wiedemann syndrome, in which specimens are often completely cystic (3, 15). PBLs are acinar neoplasms, and their microscopic features consist mostly of epithelial monomorphic polygonal cells arranged in a solid, trabecular, or acinar pattern with frequent mitoses. Squamoid nests or corpuscles are characteristic and may be scattered throughout the tumor, however, these features may not be identified on a small biopsy. Squamous corpuscles are characterized by clusters of polygonal cells or whorled spindle cells, with or without central keratinization and expression of epithelial membrane antigen and LEF1 on immunohistochemical staining (3, 8, 15, 21–23) (Figures 1A,B). PBL demonstrates positive immunostaining for markers of acinar differentiation such as trypsin, chymotrypsin, and Bcl10, though positivity for neuroendocrine markers, such as chromogranin and synaptophysin, and alpha-fetoprotein (AFP)-positive cells have also been identified (15, 21). Beta-catenin staining may also be noted, predominantly within squamoid corpuscles and in a subset of background neoplastic cells. If present, stroma is often hypercellular, containing spindle-shaped cells (8, 15).

### Clinical presentation and imaging characteristics

Patients with PBL are usually asymptomatic but will have a large palpable abdominal mass (3, 24–26). When present, symptoms are non-specific and include abdominal pain, anorexia, weight loss, vomiting, diarrhea, and fatigue (3, 7, 24, 25). Jaundice is usually not present (3, 7). AFP levels are a reliable tumor marker in 70%–80% of patients and has been shown to correlate with tumor size (5, 8, 15, 18). When elevated, serum AFP may be used in disease surveillance due to evidence of its reduction after treatment and elevation in disease recurrence (18, 27, 28).

**Figure 2:**
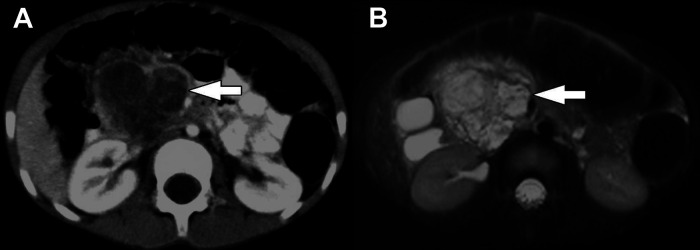
Pancreatoblastoma. (**A**) Axial CT of the abdomen obtained in the portal venous phase following intravenous contrast material administration shows a heterogeneously hypoenhancing mass arising from the head and neck of the pancreas (arrow). (**B**) T2-weighted fat-saturated MRI in the same patient shows the mass (arrow) to be heterogeneously hyperintense with the appearance of internal complexity.

PBL may be large on presentation, making it difficult to identify its origin within the pancreas. It may appear to compress nearby structures, and local invasion may not be identified until operation. Due to the soft nature of the tumor, biliary compression is rare, though reports of arterial encasement have been published. Locoregional assessment is performed using ultrasound (US), computed tomography (CT), and magnetic resonance imaging (MRI). An abdominal US is often the first modality performed in patients with non-specific symptoms and typically demonstrates a large, solitary lesion with mixed echogenicity and multiple lobulations of solid and cystic components. Cyst-dominant tumors will be more hypoechoic with hyperechoic septae (8, 16). Ultrasound is not adequate to assess the locoregional extent of disease. For this, CT or MRI are required. On multi-phase CT imaging, PBL will demonstrate heterogeneous enhancement, specifically in the septae, as well as both solid and cystic components. Calcifications may also be present in clusters or in a curvilinear distribution. On MRI, T1-weighted imaging characteristically demonstrates a well-circumscribed mass with low-intermediate signal intensity, while T2-weighted imaging demonstrates necrotic and hemorrhagic components with high signal intensity (3, 8) (Figure 2). Endoscopic ultrasound (EUS) can be used to further characterize the tumor, evaluate vascular components, and obtain tissue for diagnostic purposes.

When PBL is locally invasive, it appears as a mass with poorly circumscribed borders and may occupy peripancreatic tissues or adjacent organs. There are also limited reports of biliary and vascular invasion (8, 24). Metastasis on initial evaluation has been reported in 17%–35% of patients, with lymph nodes and liver being the most common locations (7, 29). To assess for metastatic disease, cross-sectional imaging of the abdomen with contrast-enhanced CT and/or MRI are necessary in addition to a chest CT (16).

Due to the lack of a specific staging system for PBL, an evidence-based classification has been suggested by the European Cooperative Study Group for Pediatric Rare Tumors (EXPeRT) group (16). The system, which is based on clinical and pathologic features, was created using the results of initial operation among 20 children with PBL from 2000 through 2009 in Italy, France, Germany, Great Britain, and Poland. Stage I is compatible with a completely excised tumor and an R0 resection with no evidence of pathologic lymph nodes. Stage II includes grossly resected tumors with suspected residual (R1) disease or completely resected tumors (R0) with positive lymph node(s) also completely resected. Stage III tumors are resected or biopsied with gross residual disease (R2) regardless of lymph node status. Lastly, stage IV indicates presence of metastatic disease (16, 18).

### Treatment

The most effective and mainstay treatment of PBL is complete surgical resection. It is also the most important prognostic factor, with 5-year overall survival rates of >70% reported in patients without metastases who underwent R0 resection (6, 18). In 81 pediatric cases of PBL, most patients underwent pancreaticoduodenectomy (PD; Whipple procedure) (44.1%), followed by spleen-preserving distal pancreatectomy (DP) (26.5%), central pancreatectomy (11.8%), tumor enucleation (11.8%), and DP with splenectomy (5.8%) (5). Neoadjuvant chemotherapy may be required in cases of metastases or local invasion to allow for complete surgical resection. Often, after establishing a tissue-confirmed diagnosis of PBL, cisplatin and doxorubicin are administered for 4–6 cycles (6, 8, 18, 30). Although substantial tumor regression has been reported in 50%–73% of patients undergoing neoadjuvant chemotherapy, the benefits of adjuvant chemotherapy are less clear and often utilized when surgery is incomplete (R1 or R2) or to prevent relapse (8, 16, 27–29). Radiation therapy may also be considered for incomplete surgical resection; however, clinical benefits are also currently unknown (5, 28).

Historically, 30%–60% of patients were expected to experience relapse of PBL, although more recent reports have suggested a much lower occurrence (14.7%) (5, 6, 16). In relapsed disease with metastases to the liver, prognosis is poor and management strategies are not well-defined (6). A recent meta-analysis demonstrated that surgical resection was again fundamental in successfully managing local relapse or metastases, whether *via* metastectomy or ablation (6). Three case reports were also identified in which patients with relapsing multicentric metastases to the liver were managed with liver transplantation (3 remained alive, 1 with a third recurrence). Authors also concluded that second-line chemotherapy with ifosfamide, etoposide, and a platin derivative with or without an anthracycline appeared most beneficial in making tumors amenable to a salvage resection (6). There are no established protocols in place for surveillance of PBL after treatment; however, long-term follow up is highly recommended due to the risk for recurrence. According to the classification proposed by the EXPeRT group, patients with stage II and III disease should have follow-up with imaging (CT or MRI) every 3 months in years 1 and 2 after treatment, every 4 months in year 3, every 6 months in year 4, and annually thereafter (16).

## Solid pseudopapillary neoplasm

### Epidemiology

Solid pseudopapillary neoplasm (SPN) is an exocrine pancreatic neoplasm which was first characterized in 1959 by Virginia Frantz (31). Over 700 cases have now been documented in English literature, and another 550 cases have been documented in Chinese literature. Approximately 22% to 53% have been reported in the pediatric population (3, 8, 32–35). Most are diagnosed in adolescent and young females in their 2^nd^ or 3^rd^ decade of life. Reports have demonstrated a mean age of 21.9 years at diagnosis overall and 13–14 years among pediatric populations (30, 33, 36). The cellular origin of SPN remains unknown, however, it is thought to arise from embryonal pancreatic pluripotent cells due to consistent negative staining for mucin, enzymes, and hormones (8, 37, 38).

### Gross pathology and histology

There is debate regarding the tumor's most common location, and although they can occur throughout the pancreas, a majority are described in the pancreatic tail (36, 39). There have also been isolated reports of extrapancreatic SPN occurring in the mesocolon, omentum, ovary, and retroperitoneum (40–45). Tumors range in size from <0.5 cm up to 20 cm, although most average 5 cm–7 cm in diameter (30, 33, 36, 38, 46, 47). Fortunately, SPN is a slow-growing, indolent tumor of low malignant potential (7%–16%) (3, 48). They are often solitary and ovoid, with those larger in size being sharply circumscribed and surrounded by a fibrous capsule (3, 8, 34, 49). Their cut surface can be heterogenous with soft and friable solid areas, cystic/hemorrhagic areas and necrosis (3, 49, 50) (Figure 1C). Their composition can range from solid to entirely cystic, although smaller lesions are usually solid, less circumscribed, and unencapsulated (34). Calcifications may also be present, most often within the capsule (3). Larger tumors with an increased solid composition have been associated with an increased risk of malignancy and recurrence (8, 48). More aggressive tumors also seem to arise in males and demonstrate infiltrative growth patterns. Although largely sporadic, there have been familial cases reported which also demonstrate more aggressive behavior (8, 37, 51).

Microscopically, SPNs are variably composed of solid and cystic elements with interspersed hemorrhage. The most distinctive characteristic is the presence of pseudopapillae, which are composed of central hyalinized fibrovasacular cores and surrounding layers of discohesive epithelial cells whose nuclei tend to be located away from the central fibrovascular cores (Figure 1D). Solid areas are composed of sheets of polygonal epithelial cells with clear, eosinophilic to foamy/vacuolated cytoplasm surrounded by delicate microvasculature (3, 34, 52). Nuclear grooves and extracellular hyaline globules may be frequently noted. Immunohistochemistry is a reliable diagnostic adjunct in diagnosing SPN as they can have overlapping radiographic characteristics with pancreatic pseudocysts, acinar cell carcinoma (ACC), mucinous neoplasms, and pancreatic neuroendocrine tumors (NET) (46). SPNs are characterized by diffuse nuclear beta-catenin and LEF-1 expression in addition to cytoplasmic CD10 expression (22) (Figure 1E). They are frequently positive for synaptophysin, and less frequently for S-100. SPNs lack chromogranin expression, which helps in its distinction from pancreatic NETs, in addition to the above markers (39, 46, 47, 49, 53, 54). Almost all SPN tumors demonstrate nuclear expression of beta-catenin due to an inherent mutation in the *β-catenin* gene which results in abnormal protein expression (47). Progesterone and estrogen receptors have also been identified on tumor cells, which has been proposed as an explanation for the tumor's predilection for females, although evidence is inconsistent (19, 32, 38, 52, 55). Tamoxifen has, therefore, been considered a potential therapeutic agent, although its effects are unknown (32).

### Clinical presentation and imaging characteristics

Due to the slow-growing nature of SPNs, they often go undiagnosed until reaching significant size, on average >8 cm. However, a small proportion of patients are incidentally diagnosed (46, 49). Abdominal pain is the most common symptom at presentation, and some patients may have a palpable mass or abdominal fullness (33, 34). Jaundice is less frequent but may occur if the tumor is located in the head of the pancreas causing biliary obstruction (37). AFP, CEA, and CA 19–9 are typically normal (30, 46, 47).

**Figure 3:**
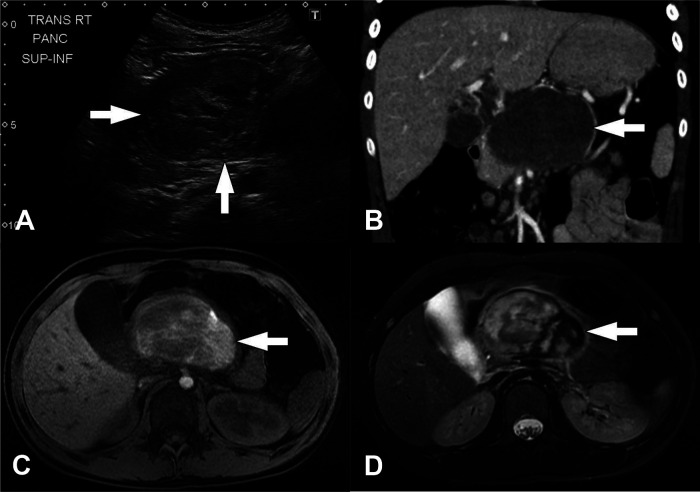
Solid pseudopapillary neoplasm. (**A**) Transverse image from initial transabdominal ultrasound shows a heterogeneous mass (arrows) in the expected location of the pancreas. (**B**) Coronal reformatted image from subsequently performed CT of the abdomen obtained in the portal venous phase following intravenous contrast material administration shows a heterogeneously hypoattenuating mass arising from the body of the pancreas (arrow). Subtle enhancing septations/material are visible within the mass. (**C**) Axial T1-weighted and (**D**) axial T2-weighted images from subsequently performed MRI show the mass in the body of the pancreas (arrows). The mass is internally heterogeneously T1-weighted and T2-weighted hyperintense with prominent peripheral T2-weighted hypointensity. These signal characteristics suggest the presence of internal hemorrhage.

US of the abdomen will typically demonstrate a well-demarcated mass with varying degrees of heterogeneity based on tumor composition, with hyperechoic and hypoechoic areas. The fibrous capsule may also be visualized (49, 56). CT will also demonstrate these findings with a hypoattenuating, or less frequently, a contrast-enhancing capsule (3, 57). Due to their large size, tumors frequently compress adjacent structures but are unlikely to be invasive. However, invasive features may be poorly identifiable on imaging, often appearing well-demarcated (58, 59). The internal structure of SPNs is complex, with varying amounts of echogenic solid components and hypoechoic cystic areas of hemorrhage. However, the fibrous capsule and internal hemorrhage are the most distinguishing features of SPN compared to other pancreatic tumors (34, 35, 44, 49, 60, 61) (Figure 3). Internal septae and peripheral intratumor calcifications have also been described in up to one-third of cases (52, 59). Fluorine-18-fluorodeoxyglucose (FDG) positron emission tomography (PET) imaging characteristically demonstrates more intense FDG uptake compared to other pancreatic tumors, specifically in the hypermetabolic peripheral capsule, while cystic and necrotic areas have poor uptake (8, 62).

The presence of SPN's characteristic internal hemorrhage is best demonstrated on MRI imaging (Figure 3). T1-weighted images show high intensity signal in areas of hemorrhage, while solid portions appear iso- or hypotense, and the fibrous capsule appears hypointense. T2-weighted images may demonstrate a dark, fibrous rim, with solid regions appearing hyperintense, and hemorrhagic areas are more variable in signal intensity. On dynamic contrast-enhanced MRI imaging, tumors have been described as having minimal enhancement in the early arterial phase with a gradual increase in later phases (61). SPNs are generally avascular in composition, pushing vessels to the periphery (57, 59).

### Treatment

Like PBL, complete surgical resection also the standard treatment of SPN. Long-term prognosis is excellent, with 10-year survival rates >95% after adequate surgical resection, even in the presence of distant metastases or disease recurrence (34, 38, 52, 63–68). SPN metastases are most frequently to the liver, lymph nodes, and peritoneum. Although metastatic disease is rare at diagnosis in pediatric patients (<10%), it is described in up to 19.5% of adults (3, 33, 34, 48, 69). Operation often includes a DP for tumors in the body or tail of the pancreas, with splenic salvage if possible, or a PD for tumors located in the pancreatic head. Operation should concomitantly include removal of any metastatic disease amenable to resection, and tumor debulking is considered beneficial even in the setting of incomplete primary or metastatic resection (14, 34, 36–38, 69, 70). Enucleation and biopsy should be avoided, as this has led to inadequate resection margins and subsequent recurrence (8, 37, 48).

There have been reports of successful and unsuccessful use of adjuvant chemotherapy for unresectable disease. However, it is not frequently administered due to the lack of evidence-based protocols (30, 71, 72). Radiotherapy has also been suggested as a potential adjunct for unresectable disease because tumors have demonstrated radiosensitivity in multiple cases (73–75). Finally, transarterial catheter embolization (TACE) has been attempted in patients with multiple metastases, although outcomes are not well understood (76). There are currently no specific guidelines established for surveillance of SPN after surgery (77, 78). However, long-term follow up is important, especially in patients with aggressive features on pathology, and because overall rates of recurrence approach 10% with tumor relapse developing more than 10 years after initial treatment in some patients (30, 38, 69, 70). In the setting of recurrence, repeat surgical resection and tumor debulking is required (33, 46, 72, 79).

## Ductal adenocarcinoma

### Epidemiology

Pancreatic ductal adenocarcinoma (PDAC) is the most common malignant pancreatic neoplasm in adults but is exceedingly uncommon in children. PDAC is rarely seen in patients under 40 years of age, and less than 1% of cases occur in patients under 20 years old (15, 80–82). In patients younger than 20, most are males (1, 15). Most PDACs diagnosed in younger patients are often linked to hereditary syndromes (15). When arising in patients with a strong family history, they are considered familial pancreatic cancers (FPC), of which 20% have *BRCA2* mutations (83–85). Other associated genetic mutations include *p16/CDKNA* (familial atypical multiple mole melanoma syndrome), *ATM* (telangiectatic ataxia), *PRSS1* or *SPINK1* (hereditary pancreatitis), *STK11/LKB1* (Peutz-Jeghers syndrome, and *MLH1/PMS1/PMS2/MSH2/MSH6* (Lynch syndrome) (15, 83, 85, 86). Common somatic mutations also occur in the *KRAS* oncogene (90%) and tumor suppressor genes *p16, TP53*, and *SMAD4* (87, 88). In addition to gene mutations, other known risk factors for PDAC are smoking, chronic pancreatitis, and high dietary fat intake (15, 89).

### Gross pathology and histology

PDACs occur most frequently in the pancreatic head, while one-third are found in the pancreatic tail (15). Tumors are solid, firm, and yellow or gray in color. They are poorly demarcated, and typically smaller at diagnosis (2 cm–3 cm). PDACs histologically contain duct-like and tubular components comprised of columnar or mucus-secreting cuboidal cells irregularly infiltrating background pancreas with characteristic perineural invasion (15). Immunohistochemical stains are non-specific, and markers expressed include cytokeratins (7, 8, 18, 19 and 20), EMA, CEA, and CA 19–9/CA125/DUPAN-2 (90, 91) and mucin markers *MUC1, MUC3, MUC4*, and *MUC5AC* (92). In 10 patients younger than 40 years old, PDAC tumors expressed cytoplasmic *MUC1* expression in 90% (80). Loss of nuclear *SMAD4* and *p16* expression has been also widely documented in a subset of tumors (88).

### Clinical presentation and imaging characteristics

Unlike other pancreatic tumors diagnosed in children, PDAC characteristically causes obstructive jaundice due to invasion within the head of the pancreas and subsequent biliary tract obstruction. Also seen are weight loss, back pain, and new-onset diabetes (15, 82). On diagnostic imaging, PDAC presents similarly in adults and in younger patients, although a study by Ivy et al. demonstrated poorer differentiation and higher prevalence of metastases at the time of diagnosis in younger patients (81, 93, 94).

**Figure 4:**
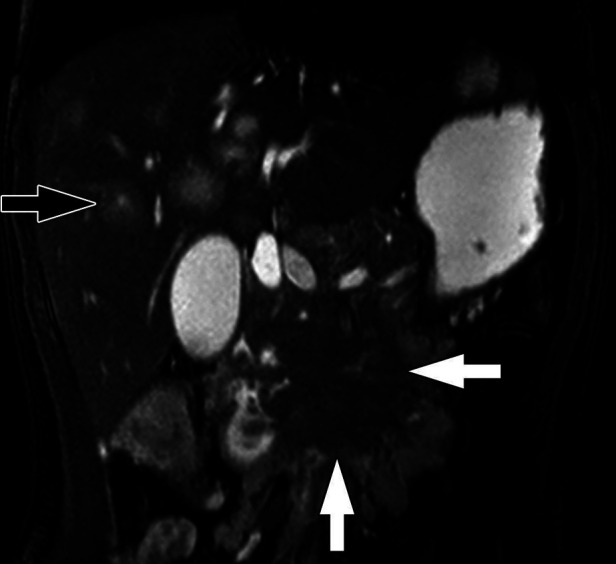
Pancreatic ductal adenocarcinoma. Coronal T2-weighted fat-saturated MRI of the abdomen shows a hypointense infiltrative mass involving the head and uncinate process of the pancreas (white arrows). There are additionally ill-defined hyperintense metastases in the liver (one indicated by the black arrow).

Overall, imaging is non-specific, with tumors varying in size with heterogeneous imaging features. Lesions may be occult by US or may appear echogenic relative to background pancreas. By CT scanning, PDAC is characteristically hypoenhancing relative to normal pancreatic tissue. MRI imaging typically demonstrates a hypointense mass on T1-weighted imaging with variable T2 signal (Figure 4). It is common for PDAC to invade adjacent structures and vasculature, specifically the biliary and pancreatic ducts, which causes ductal dilatation and the “double duct sign” on imaging (3, 8).

### Treatment

Unfortunately, greater than 50% of the reported PDACs in children are metastatic on presentation, mostly to the liver (1, 95). Like PBL and SPN, complete surgical resection is most curative in both children and adults, although only 10%–20% of PDACs are amenable to resection (82). Disease-free survival has been extended in adults using modified FOLFIRINOX (folinic acid, fluorouracil, irinotecan hydrochloride, oxaliplatin), and this regimen has also been used effectively in a small number of pediatric cases (8). Regardless, PDAC carries a poor prognosis for survival even in young patients. Published reports have demonstrated a 5-year survival rate of 4% in patients <40 years old and a 15-year survival rate of 23% in children (80, 81).

## Acinar cell carcinoma

### Epidemiology

Acinar cell carcinoma (ACC) accounts for just 2% of all pancreas neoplasms, most often diagnosed in adult men at an average age of 58 years old (96). They rarely occur in children, with 26 pediatric cases reported, although they remain more predominant than pediatric PDAC (96–100). The more common genetic alterations have been identified in *BRCA1, BRCA2, ATM, PALB2,* and *MSH2*. Chromosomal rearrangements in *BRAF* and *RAF1* have also been identified in one-fourth of ACCs (101).

### Gross pathology and histology

ACCs are found throughout the pancreas and are often large at the time of diagnosis, averaging 10 cm–11 cm in size. On gross section, they are tan to red, and fleshy. Some demonstrate areas of necrosis, hemorrhage, or cystic degeneration. They are frequently well-circumscribed and can be partially or circumferentially encapsulated (15, 96). ACCs often grow and displace adjacent structures, which is demonstrated microscopically by neoplastic cells extending as lobules through the peripheral capsule into parenchyma, vasculature, and nerves (96). On microscopic pathology, multiple growth patterns are seen – acinar, solid, glandular, and trabecular – although acinar and solid patterns are the most common. The cells often have granular and eosinophilic cytoplasm whose nuclei have a prominent nucleolus. A key distinguishing feature in comparison to PBL is that ACCs lack both squamoid corpuscles and differentiated mesenchyme (3). ACC also has scant fibrous stroma, which can help differentiate it from PDAC (8). They often stain positive for trypsin, chymotrypsin, and BCL10 on immunohistochemistry (100, 102) (Figure 1F).

### Clinical presentation and imaging characteristics

Similar to most pancreatic tumors, ACC presents with non-specific gastrointestinal symptoms (abdominal pain, emesis, and diarrhea), although weight loss may also occur (96). Unique to ACC is that 10%–15% of patients may develop lipase hypersecretion syndrome. This causes large amounts of lipase to be released into the bloodstream, exceeding 10,000 U/dl in some cases, and leads to subcutaneous fat necrosis, eosinophilia, and polyarthralgia (103, 104). Most frequently, this is seen in the setting of very large primary tumors or metastatic disease (98). Lipase may also be elevated outside of lipase hypersecretion syndrome, and AFP elevation has been reported in younger patients (98, 105, 106).

**Figure 5:**
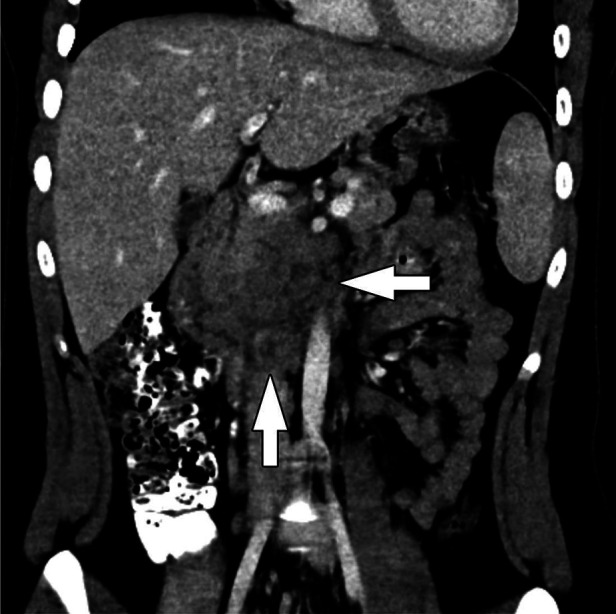
Acinar cell carcinoma. Coronal CT of the abdomen obtained in the portal venous phase following intravenous contrast material administration shows an infiltrative, hypoenhancing mass involving the head and uncinate process of the pancreas with adjacent conglomerate pathologic lymph node enlargement (arrows).

On US, tumors are usually hypoechoic and are variably defined. CT typically demonstrates a well-demarcated exophytic (on the basis of size) mass with partial or complete encapsulation (Figure 5). Tumors are characterized by internal heterogeneity and may have central hypoattenuation/hypoenhancement corresponding to necrosis. One-third of tumors will demonstrate calcifications located centrally or peripherally. ACCs show greater contrast enhancement than PDACs, but less than normal pancreatic tissue. Tumors smaller in size demonstrate more homogeneous enhancement, while larger tumors demonstrate enhancement of their solid components in the periphery (3, 107). MRI findings are less described for ACC but in 2 patients, T1-weighted imaging showed hyperintense signal in one mass and a central mixed signal in the other. Both were hyperintense to pancreatic tissue on T2-weighted images (107). Although the appearance of ACC on imaging can resemble PBL and SPN due to their large size and characteristic central necrosis, PBL and SPN are much more common in children (3).

### Treatment

ACC is an aggressive tumor with >50% of patients having metastatic disease on presentation, most commonly to liver and lymph nodes (97, 98). Complete surgical resection with or without the addition of chemotherapy has been shown to provide the best outcomes. Previous results demonstrate 5-year survival rates of 36% in patients with resected disease, compared to 10% in patients who did not undergo resection (108). Although more dismal than most pancreatic tumors, ACC does have a better prognosis than PDAC (96). Fortunately, more recent studies have demonstrated 5-year survival rates of up to 50% for localized disease, compared to older studies which suggested 5-year survival of 6% (108–110). For unresectable disease, only a very small number of patients who received chemotherapy and/or radiation experienced a significant response (104, 109).

## Neuroendocrine tumors

### Epidemiology

Pancreatic neuroendocrine tumors (NET), also known as islet cell tumors, account for 2% of all pancreatic neoplasms and 5%–10% of all pediatric pancreatic tumors (111, 112). These tumors derive from pancreatic islet cells and are frequently distinguished as well-differentiated, benign adenomas or poorly differentiated, metastatic carcinomas (3, 113). In children, 90% of pancreatic NETs are benign. The overall mean age at diagnosis in all pancreatic NETs is 47 years old, and most pediatric cases are diagnosed in older children (3). Genetic predispositions may also be present, specifically in pediatric cases, with approximately 5% of pancreatic NETs being associated with multiple endocrine neoplasia type 1 (MEN-1). Genetic associations are also seen in Von-Hippel Lindau (VHL) disease, neurofibromatosis type 1 (NF-1), and tuberous sclerosis complex (TSC) (114, 115). Another important distinction made in pancreatic NETs is whether the tumor is functioning, which means active hormones are secreted by islet cells to produce clinical symptoms, or it is non-functioning/clinically silent (3). In children, the most common pancreatic NETs are insulinomas and gastrinomas (3).

#### Insulinoma

Derived from beta cells, insulinomas peak in incidence between 40 and 60 years old (113). Although they remain the most common pediatric pancreatic NET, they rarely occur before age 15, with 4.9% being found in children aged 10–19 years old and just 0.9% found in 0–9 year olds (116, 117). Patients with MEN-1 are diagnosed at a mean age of 27 years old, which is 20 years younger than patients without the genetic association (117). Insulinomas are found primarily in the pancreas, although some reports of primary extrapancreatic lesions in the duodenum, ileum, lung, cervix, and ovary have been made (118–123). Due to uncontrolled insulin secretion, insulinomas are associated with clinical symptoms of fasting hypoglycemia, palpitations, perspiration, tremors and even seizure. Persistent hyperinsulinemic hypoglycemia (PHH) may occur, causing mental confusion, fatigue, weakness, and seizures and resolves with the administration of glucose (113). In younger children, these classic symptoms may instead present as behavioral changes, seizures, and coma. Unfortunately, untreated persistent hypoglycemia may lead to lasting neurologic effects (14). Most insulinomas are benign, solitary lesions which are small in size (∼2 cm) (123, 124). However, of the 10% which are malignant, tumors are often greater than 2 cm in size, and one-third of patients are reported to have metastatic disease at the time of presentation (124, 125).

#### Gastrinoma

Gastrinomas are derived from gastrin-secreting G cells. Their peak incidence is from 48 to 55 years old, and as seen in insulinomas, children 5–15 years old are rarely affected (14, 113). They are most commonly located within the gastrinoma triangle, which is comprised of the head of the pancreas, the first and second portions of the duodenum, and the porta hepatis (14, 113). Tumors are typically solitary, though when multiple gastrinomas are present, Zollinger-Ellison syndrome (ZES) should be considered. Clinically, they cause hypersecretion of hydrochloric acid in the stomach, leading to peptic ulcer disease, gastroesophageal reflux disease (GERD), and most commonly, diarrhea. However, these symptoms may be less pronounced than those seen with hyperinsulinemia, and an accurate diagnosis may be delayed (113, 126). In contrast to insulinomas, gastrinomas are larger, with a mean size of 4.2 cm at presentation, and 60% demonstrate malignancy (14, 127).

#### Other functioning and non-functioning pancreatic NETs

Other functioning and non-functioning pancreatic NETs are either rare or unreported in the pediatric population (3). Glucagonomas are derived from alpha cells and commonly occur in the distal pancreas. Often, they are malignant (60%–70%) and larger in size, averaging 7.2 cm (14, 128). When clinically evident, glucagon secretion may cause a skin rash called necrolytic migratory erythema, which is seen in up to 80% of patients. Glucose intolerance, weight loss, depression, and the tendency to develop deep venous thrombosis may also be seen (129). Somatostatinomas derive from D cells. Patients may present with symptoms of cholelithiasis, steatorrhea, diabetes mellitus (DM), and hypochlorhydria due to the repressive nature of the somatostatin hormone (14, 130). Thus far, there have been no reports of pancreatic glucagonoma or somatostatinoma in children (3, 131). Another uncommon functional pancreatic neuroendocrine tumor is VIPoma, which is composed of D1 cells, secretes vasoactive intestinal peptide (VIP), and causes large volume diarrhea, hypokalemia, and achlorhydria. Most frequently, pediatric cases of VIPoma are not reported in the pancreas (125).

Non-functioning pancreatic NETs are hormonally inactive or clinically silent, often causing a more delayed diagnosis in comparison to symptomatic, functioning tumors. Mean age at presentation is 70 years old, with most tumors averaging 2 cm–5 cm in size and demonstrating malignant features. When symptoms are present, they are typically due to local invasion and mass effect (3). Nearly 10% demonstrate no immunohistochemical staining for pancreatic hormones, although others express glucagon, pancreatic polypeptide (PP), somatostatin, serotonin, and/or calcitonin. Without a hormonal syndrome, they are not considered functioning pancreatic NETs (14, 98, 132).

### Gross pathology and histology

Pancreatic NETs are typically round and well-demarcated, with a consistency ranging from soft to firm. Histology of pancreatic NETs is also variable, often with sheets of monomorphic cells which are arranged in a trabecular, acinar, pseudoglandular or solid pattern (3). Nuclei are often round-oval with speckled chromatin. Immunohistochemistry is extremely helpful in establishing the neuroendocrine properties of pancreatic NETs, especially chromogranin, synaptophysin and CD56. Grading is often determined using the World Health Organization's classification which is based on mitotic rate and Ki-67 proliferation (133, 134).

### Imaging characteristics

**Figure 6:**
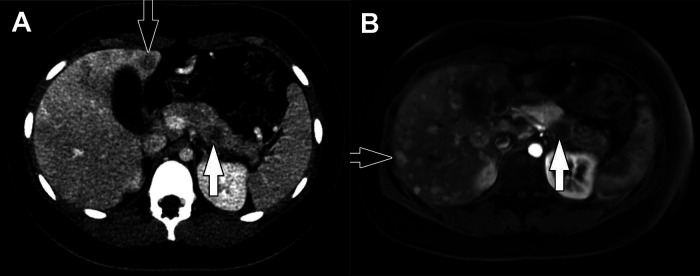
Insulinoma. (**A**) Axial CT of the abdomen obtained in the portal venous phase following intravenous contrast material administration shows a hypoenhancing mass in the body of the pancreas (white arrow) with multiple hypoenhancing liver metastases (one indicated by the black arrow). Characteristically, neuroendocrine tumors hyperenhance in the arterial phase following contrast administration but hypoenhancing lesions do occur. (**B**) Axial T1-weighted fat-saturated post-contrast MRI obtained in the arterial phase shows the pancreatic tumor (white arrow) to be hypoenhancing while the multiple liver metastases (one indicated by the black arrow) are hyperenhacing. The tail of the pancreas upstream of the tumor is hypoenhancing due to obstructive pancreatitis related to the tumor.

**Figure 7:**
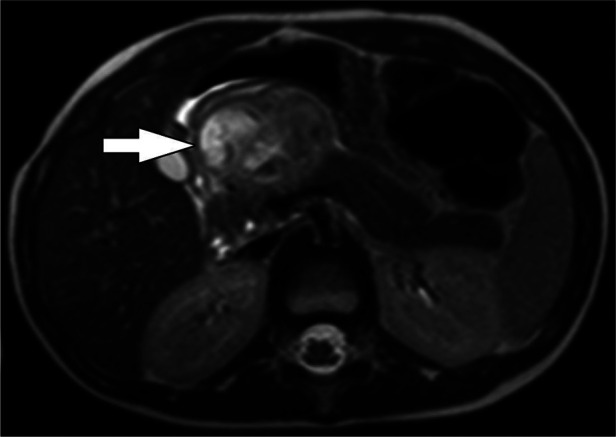
Pancreatic NET. Axial T2-weighted MRI shows a heterogeneously hyperintense exophytic mass arising from the neck of the pancreas (arrow). The upstream pancreatic body and tail are Normal and there is no duct dilation to suggest duct obstruction.

Most pancreatic NETs are insulinomas and are typically small, homogenous, and well-circumscribed on imaging. Gastrinomas and other functioning and non-functioning pancreatic NETs may be larger and more heterogeneous (126, 127). On US, insulinomas are typically round and hypoechoic, possibly with a hyperechoic rim, while larger pancreatic NETs may demonstrate cystic areas or calcifications (127). Multi-phasic CT or MRI are the standard imaging modalities used to diagnose pancreatic NETs (135, 136). Pancreatic NETs characteristically show homogeneous, hyperintense enhancement on CT or MRI following contrast administration (137) (Figures 6, 7). Larger, malignant pancreatic NETs are often heterogeneous with non-enhancing cystic components and solid enhancing components located at the periphery (127). On MRI, lesions are usually hypointense on T1 weighted imaging and hyperintense on T2 weighted imaging (8, 138).

As with other pancreatic tumors which are being evaluated for potential enucleation, EUS may be utilized if CT and/or MRI are not diagnostic (139). It has been shown to be highly accurate in endocrine tumor localization; however some studies have also demonstrated that EUS is more operator-dependent, significantly affecting accurate detection (140–143). Additional imaging adjuncts like angiography, transhepatic portal venous sampling, intra-arterial calcium-stimulated venous sampling, and somatostatin receptor scintigraphy have also been utilized for pancreatic NET localization in hormonally active tumors, although these remain less sensitive than CT and MRI (3, 135–137, 144–146). Somatostatin receptor scintigraphy can be helpful for when tumors express somatostatin receptors, which occurs in 60%–70% of insulinomas (3). Indium-111 (^111^In) octreotide SPECT/CT has been replaced by [^68^Ga]-DOTA-TATE PET/CT, as the newer somatostatin analogue (^68^Ga-DOTA-tyrosine^3^-octreotide) has shown higher detection rates, while remaining low in toxicity and radiation exposure (147).

### Treatment

Typically, complete surgical resection is performed to obtain curative treatment for pancreatic NETs. Tumors in the pancreatic head will be resected with a PD, while those more distal in the pancreas will undergo a DP; however, gland-preserving procedures like duodenum-preserving pancreatic head resection (DPPHR), central pancreatectomy, and tumor enucleation are also performed based on the function of the pancreatic NET, its size, and its benign vs. malignant characteristics (36, 148). Enucleation may be especially useful for benign pancreatic NETs like insulinoma because it provides desirable outcomes even when margins are positive. Some functioning pancreatic NETs may also require symptom control prior to resection, typically with octreotide (149). Providers may consider observation and active surveillance in small non-functioning tumors, as just 6% have been identified as malignant in previous reports, although evidence remains limited especially in pediatric patients (148). In the presence of unresectable, locally advanced, or metastatic disease, chemotherapy (a combination of temozolomide-capecitabine, everolimus, or sunitinib) or radionuclide therapy with lutetium-177 (^111^Lu)-DOTA-TATE may be used (150). Like [^68^Ga]-DOTA-TATE for tumor localization, [^111^Lu]-DOTA-TATE targets the somatostatin receptor for therapy. Prognosis varies based on tumor type, histology, and risk for malignancy. Although most benign insulinomas are cured by surgical resection, the median survival of patients with metastatic disease is <2 years (117, 151). Gastrinomas without metastatic disease have a 10-year survival rate of 90%–100%, while most other functioning pancreatic NETs have a 5-year survival rate of less than 50% due to tumors often being advanced at diagnosis (152, 153). In children specifically, reports have demonstrated 15-year survival rates of 50% across pancreatic NETs overall (1).

## Non-epithelial tumors

### Pancreatic Ewing sarcoma

Pancreatic Ewing sarcoma (formerly, primitive neuroectodermal tumor), accounts for just 0.3% of pancreatic neoplasms (154). Typically, they are an aggressive tumor affecting bone, but nearly 30 pancreatic Ewing sarcomas have been reported in patients under 25 years old (155, 156). Mean age at diagnosis is 18.2 years, and there is no sexual predominance (156). The characteristic genetic translocation involves the *EWSR1* gene on chromosome 22q12 (155) and has been recognized in several reported cases of pancreatic Ewing sarcoma. However, Ewing sarcomas can also demonstrate rearrangements involving the *FUS* gene (157). Over two-thirds of pancreatic Ewing sarcomas are diagnosed within the pancreatic head, ranging in size from 3.2 to 22 cm. Histological characteristics include clusters or nests of small, round blue cells with scant cytoplasm to moderate amounts of clear to eosinophilic cytoplasm. Cells express the product of the *MIC2* gene, which is confirmed by strong diffuse membranous positivity for CD99 on immunohistochemistry (156). NKX2.2 is a nuclear immunohistochemical stain that is also helpful in diagnosis (158).

Abdominal pain is the most common symptom at presentation, followed by jaundice and nausea. Although the tumor can reach significant size, its growth is expansive vs. invasive, causing less obstructive jaundice than expected (156). On imaging, lesions are poorly defined, with cystic or necrotic areas, and heterogeneous enhancement. CT imaging demonstrates a mass hypo- or isointense to normal pancreatic tissue, and MRI imaging demonstrates an iso- or hyperintense mass on T2-weighted imaging. The tumor is also metabolically avid on FDG CT/PET imaging (3, 8).

Complete surgical resection, frequently in combination with chemotherapy (cisplatin, doxorubicin, and high-dose methotrexate), produces the best patient outcomes due to the aggressive nature of pancreatic Ewing sarcoma (159). In cases in which complete resection is not possible, radiation may help control some disease progression (156). Overall pancreatic and extra-pancreatic 5-year survival outcomes are 55%–65% with localized disease and a multimodal approach (160).

### Lymphoma (non-Hodgkin's, Burkitt)

Lymphomas involving the pancreas are extremely rare and may originate from distant lymph nodes, tumor extension from peripancreatic lymphadenopathy, or as primary pancreatic lymphoma (PPL). PPL accounts for less than 2% of extra-nodal lymphomas and occurs most frequently in men in their 5th or 6th decade of life. In children, the average age at diagnosis is 10.3 years, ranging from 3 to 16 years old (161). A majority are non-Hodgkin's lymphoma of B-cell type, specifically, Burkitt lymphoma and diffuse large B-cell lymphoma (162). Non-Hodgkin's lymphoma is also thought to be the most common pediatric pancreatic tumor of non-epithelial origin. A previous study demonstrated that one-third of pediatric patients with non-Hodgkin's lymphoma were found to have pancreatic involvement at autopsy (163). Often, it is distinguished from other tumor types by its large, multiple nodal masses; however, Burkitt lymphoma may present as a solitary lesion, multiple masses, or diffuse infiltration, mimicking acute pancreatitis (162, 163). Overall, PPL tumors in children average 5 cm–6 cm in size at diagnosis, and have a predilection for the pancreatic head (161). Histologically, cells have large lymphocytic nuclei, prominent nucleoli, and background necrosis (162).

**Figure 8:**
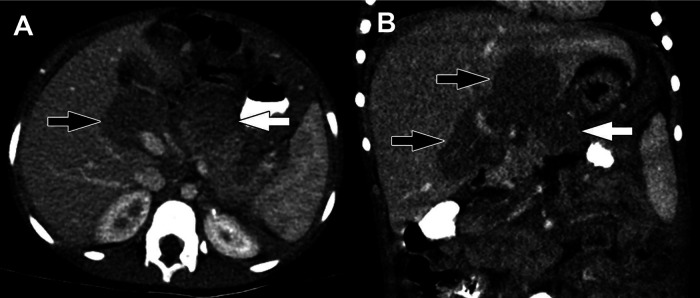
Burkitt lymphoma. (**A**) Axial and (**B**) Coronal reformatted images from a CT of the abdomen obtained in the portal venous phase following intravenous contrast material administration show hypoenhancing masses in the pancreas (white arrows) and porta hepatis (black arrows). Multiorgan involvement and infiltrative growth are highly suggestive of Burkitt lymphoma.

Commonly, patients present with abdominal pain, fevers, night sweats, weight loss, and jaundice. In patients with bulky disease in the pancreatic head, gastric outlet and duodenal obstruction may also occur (162). On US, disease appears hypoechoic relative to normal pancreas. CT imaging demonstrates either focal or homogeneous enlargement of the pancreas and patchy hypoenhancement (Figure 8). MRI will similarly show hypoenhancement of tumor involved parenchyma or lymph nodes (3). Lymphoma is optimally staged by FDG CT/PET which shows intense avidity (162).

Standard treatment of PPL is chemotherapy, which not only controls symptoms but provides long-term tumor resolution. Cyclophosphamide, doxorubicin, vincristine, and prednisone (CHOP) are frequently used, with the addition of rituximab for diffuse large B-cell lymphoma due to improved response rates. Surgery is reserved for patients with biliary obstruction, gastric or duodenal obstruction, or when the diagnosis is unclear (162). After immunochemotherapy and/or surgery, 15 pediatric patients with follow-up data reported were all alive and had reached complete remission at a median follow up of 56 months (161).

### Lymphatic malformations (lymphangioma)

Pancreatic lymphangiomas, or lymphatic malformations (LM), account for just 0.2% of pancreatic lesions and less than 1% of LMs overall (164–166). Sixty cases have been reported in the literature thus far (167, 168). They are reported across all ages and are more common among women (166). LMs are often congenital masses which develop due to lymphatic obstruction during gestation. If located within the pancreatic parenchyma, adjacent to the gland, or connected by a pedicle, they are considered to originate from the pancreas (169). They are typically slow growing, benign, and occur throughout the pancreas, with sizes ranging from 3 cm to 20 cm (166). Pathologically, pancreatic LMs are multicystic masses with a thin, fibrous capsule. Their micro- and macrocystic regions contain serosanguinous or chylous fluid (3). Histology reveals endothelial cells lining the cyst walls, smooth muscle, collagenous connective tissue and scattered lymphoid aggregates (166).

**Figure 9:**
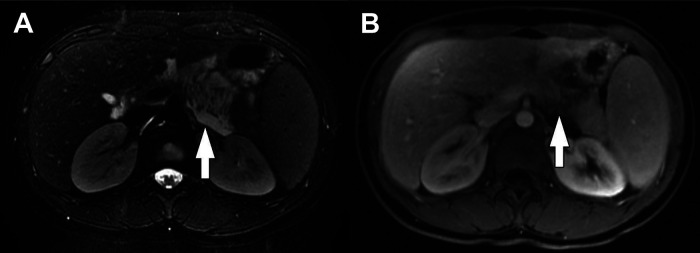
Lymphatic malformation. (**A**) Axial T2-weighted fat-saturated MRI and (**B**) Axial T1-weighted fat-saturated post-contrast MRI show an infiltrative lesion involving the body of the pancreas (arrows). The lesion is T2-weighted hyperintense reflecting fluid content without enhancement following contrast administration. Transspatial involvement is characteristic of a lymphatic malformation.

Most patients with pancreatic LMs are asymptomatic and diagnosed by incidental imaging findings, while others may endorse non-specific gastrointestinal symptoms due to mass effect. Acute presentations have also been reported related to pedicle torsion and rupture (170). On imaging, lesions are characteristically trans-spatial and can either appear as well-defined cystic lesions or more infiltrative fluid collections. Fluid content is characteristically simple (by US and CT) and hyperintense on T2-weighted MRI (Figure 9). Lesions complicated by hemorrhage may have more complex fluid content. A thin capsule and fine internal septations may be apparent and generally enhance following contrast administration. Microcystic regions will appear more solid and enhancing (171). Although imaging is useful, differentiating LMs from other cystic pancreatic lesions and pancreatic or peripancreatic fluid collections can be difficult, and EUS with fine needle aspiration/biopsy and histologic evaluation are often necessary to confirm the diagnosis (166).

Although benign, complete surgical resection is often indicated due to the tumor infiltrating adjacent organs, growing and causing mass effect, or leakage of lymphatic fluid. This may require simple excision of the lesion or larger pancreatic resections (172). However, in asymptomatic patients without local invasion, surveillance imaging is considered appropriate (166).

### Kaposiform hemangioendothelioma

Kaposiform hemangioendothelioma (KHE) is an infiltrative vascular tumor known for its aggressive nature and occurrence during infancy or early childhood (173). Although the tumor is most frequently cutaneous in origin, rare occurrences have been reported in the pancreas. It has been estimated to affect less than 1 in 100,000 children, with less than 10 pediatric cases documented in the literature (173–177). Histologically, it is composed of infiltrating nodules, sheets of spindled endothelial cells, and slit-like vascular channels. Microthrombi and hemosiderin deposits are also seen (178). Immunohistochemistry is positive for lymphatic endothelial markers but negative for glucose transporter protein type 1 (Glut1), which distinguishes KHE from infantile hemangioma (174, 177, 179).

Presenting symptoms are typically related to obstructive jaundice and increasing abdominal distention (174, 176). A key clinical feature of KHE is its association with Kasabach-Merritt phenomenon (KMP), which occurs in up to 70% of patients. Tumors with this association are typically >8 cm in size (180). The phenomenon is characterized by consumptive coagulopathy, hemolytic anemia, and thrombocytopenia. In these patients, platelet transfusions should be avoided as they can cause painful engorgement of the lesion (177, 178). KHE often appears as a homogenous soft tissue mass on US, while CT and MRI show a poorly defined mass with infiltration of surrounding tissues and heterogenous contrast enhancement (175).

Although complete surgical resection is ideal, the infiltrative growth pattern of KHE often makes this difficult, requiring excision and adjuvant chemotherapy. Sirolimus, vincristine, interferon, steroids, aspirin, and ticlopidine have been used in clinical practice but with varying outcomes of treatment (175, 176, 181). Mortality rates of 12%–24% have been reported, and typically occurs in patients with KMP (180).

### Dermoid cyst

Dermoid cysts of the pancreas, also known as mature teratomas, are benign and extremely rare, with just 35 known cases reported in the literature. Just 8 of those patients were children (182, 183). Due to their development at the time of neural groove closure, extragonadal dermoid cysts are most often found along the midline, although a majority of pancreatic dermoid cysts are found in the pancreatic head. They are often large, reported from 8 cm–12 cm in size. Cysts are composed of tissue from all three germ layers and are a combination of cystic and solid structures such as teeth, hair, cartilage, and dermal contents (3, 183).

In pediatric cases, the most common presenting symptom is vomiting; however, abdominal pain and back pain may also occur. A pre-operative diagnosis can be challenging, and the pathognomonic finding of fat/fluid or hair/fluid levels only occurs in a small number of pancreatic cases (184). Adjunctive EUS has also been used for diagnosis, but tumor appearance varies across reports (185). On CT imaging, cysts are round and well-circumscribed. They are very hypodense and heterogenous, with varying cystic and solid ratios depending on their composition (3, 183, 184). In most cases, simple excision of the pancreatic dermoid cyst is performed, although they sometimes require a DP, or less commonly, a PD (183).

### Inflammatory myofibroblastic tumor

Inflammatory myofibroblastic tumors (IMT) are rare mesenchymal tumors of unknown origin most commonly occurring in the lungs of children and adolescents (186). However, they have also been described in the head/neck, liver, pancreas, thyroid, and genitourinary tract (187–191). There have been 14 reported cases of pancreatic IMT in children, ranging from ages 6 months to 15 years old, but with a mean age at diagnosis overall of 42 years. Pancreatic IMTs are low-grade, slow-growing solid tumors located in the head or tail of the pancreas (186, 192). Microscopically they demonstrate myofibroblastic spindle cells with varying proportions of plasma cells, mast cells, eosinophils, lymphocytes, and histiocytes (193). Nearly 50% of IMTs demonstrate rearrangements in *ALK* gene, while rearrangements involving *ROS1, PDGFRβ*, *RET* and *NTRK* have been reported in the *ALK*-negative subset. With the identification of gene rearrangements novel targeted therapies are an option for unresectable tumors (194).

**Figure 10:**
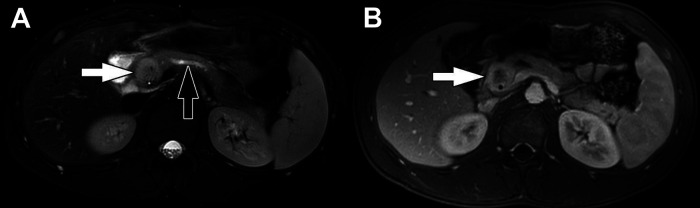
Inflammatory myofibroblastic tumor. (**A**) Axial T2-weighted fat-saturated MRI and (**B**) Axial T1-weighted fat-saturated post-contrast MRI show a mass in the head of the pancreas (white arrows). The mass is heterogeneously T2-weighted hyperintense with heterogenous enhancement following contrast administration. Lack of central enhancement suggests necrosis. There is associated dilation of the upstream pancreatic duct (black arrow) due to obstruction by the mass.

Clinical symptoms are frequently non-specific, though obstructive jaundice may occur in the setting of a pancreatic head lesion (186). Spontaneous splenic rupture has also been reported secondary to the obstruction and congestion of splenic vessels by a pancreatic tail IMT (195). By US, lesions are characteristically hypoechoic. By CT, the imaging appearance of IMTs is variable with lesions appearing hypo- or hyperenhancing with or without calcifications and necrosis. On MRI, IMTs are typically hyperintense on T2-weighted images, and on FDG PET/CT, some tumors are hypermetabolic (8) (Figure 10). IMTs have a favorable prognosis after complete surgical resection, with no reported recurrence in these patients. Radical resection is often performed, although enucleation has recently been reported, resulting in current disease-free survival (186). Radiation, chemotherapy, and corticosteroid therapy have been implemented in patients with unresectable disease or findings of malignant pathology after resection (196–198).

## Current challenges in management

### Distinguishing autoimmune pancreatitis from pancreatic carcinoma

#### Epidemiology, histopathology, clinical presentation, and imaging characteristics

Autoimmune pancreatitis (AIP) was first recognized in 1961, although a more detailed description of its histopathology was reported in 1991 (199, 200). There are currently two main types (Type 1 and Type 2) acknowledged in clinical practice and within the literature. This may or may not present as a focal pancreatic mass; however, it often causes obstructive jaundice and chronic pancreatitis (201). Studies in Japan have suggested an incidence of 1.4 per 100,000 people and found that 5%–6% of patients with chronic pancreatitis had the underlying etiology of AIP (202). In children, evidence is limited to case reports and case series, although a study in 2017 identified 48 pediatric patients in the literature and from multiple pediatric databases. Cases were diagnosed in children 2 to 17 years old, with a mean age of 13 years (203).

Type 1 AIP, known as lymphoplasmacytic sclerosing pancreatitis (LPSP), is associated with elevation of serum immunoglobulin G4 (IgG4) (201). There are also associations with extrapancreatic IgG4 disease, including Sjogren's syndrome, primary sclerosing cholangitis, inflammatory bowel disease (IBD), and rheumatoid arthritis (RA) (204). Solitary or multiple extrapancreatic fibro-inflammatory lesions may be present, and have been reported in nearly every organ system (204). Males are two times more likely to develop Type I AIP, and although all age groups are affected, it occurs most commonly in 50–60 year olds (15). On histology, it is characterized by lymphoplasmacytic infiltration of smaller interlobular pancreatic ducts, obliterative phlebitis, and peri-ductal and venous fibrosis, which mostly affects the adipose tissue within the pancreas (15, 162). In the setting of obstructive jaundice, the diagnosis of Type I AIP can often be established with a serum elevation of IgG4 greater than 135 mg/dl, which may differentiate the lesion from a more concerning PDAC (205). An elevated plasma cell ratio of IgG4 to IgG greater than 40% and immunohistochemical staining with increased positivity of IgG4 cells (>10 cells per high-power field) may also be present (179, 206, 207). In children, elevated serum IgG4 is less useful, with only 22% of patients having been reported to have IgG4 levels above the upper limit of normal (203). Mayo Clinic also put forth the HISORt criteria, which identifies five cardinal features of Type I AIP for definitive diagnosis. These features include: 1) histology suggesting lymphoplasmacytic infiltrate with storiform fibrosis, 2) imaging demonstrating a diffusely enlarged pancreas, 3) serology demonstrating elevated IgG4 levels, 4) extrapancreatic organ involvement, and 5) disease response to steroid therapy (208, 209).

Type 2 AIP, known as idiopathic duct-centric pancreatitis (IDCP), has a more elusive diagnosis due to the absence of elevated serum IgG4. In the United States, Type 2 accounts for 20 to 40% of AIP cases (202). IBD is typically the only autoimmune association and is seen in close to 30% of patients. Compared to Type 1 AIP, younger patients are more affected by Type 2, with a mean age of 43 years at diagnosis. Although periductal lymphoplasmacytic infiltrates are also present, these are typically devoid of IgG4 plasma cells. In addition, the extent of fibrosis and phlebitis is less, and there is presence of neutrophilic infiltrates of the ductal epithelium and lumen (referred to as granulocytic epithelial lesions) (15, 179, 207). Often, the definitive diagnosis of Type 2 AIP is established by histopathology review of specimens from patients undergoing surgical resection for presumed malignant disease. Otherwise, accurate diagnosis requires the use of a thorough patient history, cross-sectional imaging, endoscopic imaging, and serology (162).

Although the variants of AIP have different histopathology, their clinical and radiologic characteristics overlap with one another, as well as with concerning solid pancreatic tumors. When AIP affects the pancreatic head, strictures are formed in the distal common bile duct causing obstructive jaundice. Therefore, patients will present with painless jaundice as well as weight loss, abdominal pain, and glucose intolerance, which is similar to the presentation of PDAC. Children with AIP most commonly present with abdominal pain (90%), followed by obstructive jaundice (42%), and weight loss (29%) (203).

While the diagnosis of AIP has proven to be challenging, fewer cases are being identified on surgical pathology, likely indicating that AIP is commonly being diagnosed without surgical resection (162). US may first be the first modality to suggest AIP, demonstrating a hypoechoic, enlarged pancreas or mass-like lesion in the pancreas (8, 203). Classically, CT and MRI imaging demonstrate a diffusely enlarged pancreas, described as being “sausage-shaped” with a smooth outline due to the absence of pancreatic clefts (up to 50%–70% of adults); however, focal and multifocal enlargement may also be seen (8, 162, 210). Although Type 1 and Type 2 AIP appear similar on imaging, Type 2 disease is often more focal (85%). Delayed enhancement is demonstrated in the presence of underlying pancreatic fibrosis, and a hypoattenuating halo is present due to associated fluid, phlegmon, or fibrosis (211). MR cholangiopancreatography (MRCP) displays long strictures (defined as >1/3 the length of the main pancreatic duct), multiple strictures, or segmental/focal narrowing. Endoscopic retrograde cholangiopancreatography (ERCP) has the ability to identify characteristic ductal changes seen in AIP; however, its use is often therapeutic in cases of ductal obstruction (202). In children, cross-sectional imaging demonstrates focal gland enlargement in a slight majority of patients (52%). MRCP imaging demonstrates main pancreatic duct irregularity in 63% of patients, common bile duct (CBD) stricture/tapering in 54%, CBD dilatation in 52%, and a hypoattenuating halo in 16% (203). EUS is more commonly utilized now in diagnosis of AIP in children with high sensitivity, and it also allows biopsy of the lesion with FNA or core biopsy to allow tissue diagnosis.

#### Distinguishing autoimmune pancreatitis from pancreatic malignancy

**Figure 11:**
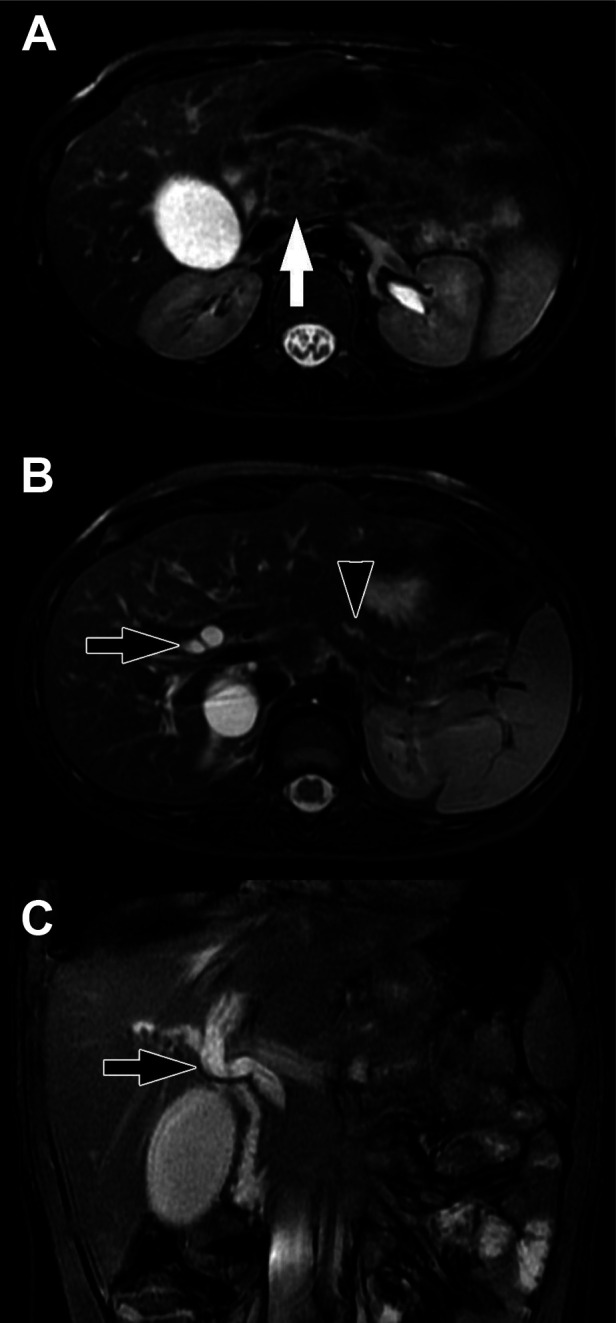
Focal pancreatitis. (**A**) Axial T2-weighted fat-saturated MFI shows heterogenous enlargement of the head of the pancreas (arrow). There is no substantial peripancreatic inflammation. (**B**) Axial T2-weighted fat-saturated MRI more cephalad in the same patient shows upstream bile duct dilation (black arrow) and dilation of the pancreatic duct (black arrowhead) due to obstruction by the process in the head of the pancreas. (**C**) Coronal balanced MRI in the same patient shows the extent of dilation of the bile ducts. The double duct sign of dilated pancreatic and bile ducts raises suspicion for a mass in the head of the pancreas.

Similarities between AIP and pancreatic carcinoma on imaging findings have led to unnecessary radical pancreatic resections in patients with AIP (15). In a study of patients in Japan undergoing PD for a pancreatic head mass from 1992 to 2005, 4% of patients were found to have AIP (202). Key characteristics seen on CT and MRCP imaging which may delineate AIP from PDAC include focal stricture or narrowing and a “capsule-like halo” with delayed enhancement (8, 210). PDAC is more frequently associated with significant pancreatic ductal dilatation and a hyperdense rim on non-contrast imaging. Importantly, focal pancreatitis in the setting of AIP can also produce both upstream pancreatic ductal dilation and CBD dilation, producing a “double duct sign” (Figure 11). Also important to recognize is that AIP may respond to a short course of corticosteroid treatment, while PDAC will not (202).

PET imaging is not advantageous in differentiating PDAC from AIP due to diffuse and intense uptake of FDG in areas of pancreatic inflammation. However, if extrapancreatic organs associated with Type 1 AIP have avid FDG uptake, this may help guide a diagnosis (202). In contrast, EUS with tissue biopsy has been shown to be especially beneficial in providing a definitive diagnosis of AIP. EUS findings include an enlarged pancreas with echogenic interlobular septa and a narrowing in the main pancreatic duct. To obtain a tissue diagnosis of AIP, EUS fine-needle aspiration may be performed. With advances in spring-loaded biopsy needles with rapid motion, adequate samples have been reported in up to 80% of cases (212). Accuracy using EUS Tru-Cut needle biopsy is even higher (85%), although technically more challenging. Algorithms have suggested the attempt at EUS fine-needle biopsy first, and if results are negative for PDAC in a case of presumed AIP, EUS Tru-Cut needle biopsy should be secondarily performed (213).

#### Treatment

The main goals of treatment in AIP are relief of symptoms and pancreatic tissue preservation. High-dose corticosteroids are the most common and successful treatment in cases of AIP, with therapeutic responses seen on imaging as early as 2 weeks (15). Typically, resolution of pancreatic inflammation, swelling, and surrounding fluid/phlegmon occurs. However, pancreatic fibrosis is long-lasting and may result in endocrine and exocrine insufficiency requiring pancreatic enzyme replacement therapy. Relapsing disease has also been reported in up to 53% of patients after steroid treatment and taper, although is more common in Type 1 AIP (60%) than Type 2 (5%) (214, 215). In a cohort of 48 pediatric patients with AIP, most were treated with steroids (60%), followed by biliary and/or pancreatic stenting (17%), partial pancreatectomy (6%), PD (4%), and choledochoduodenostomy (2%), while 17% were clinically monitored. Twenty-one percent of children experienced AIP relapse and 16% experienced exocrine insufficiency requiring enzyme replacement (203). In steroid-resistant or relapsing disease, immunomodulators and rituximab have been utilized (216). A response to steroid therapy is one of the cardinal features in the diagnosis of AIP, and failure of symptom or imaging resolution requires a prompt investigation for alternative diagnoses.

### Parenchyma-sparing resection: how much is enough?

#### Approaches to resection

When approaching pancreatic resection in pediatric patients, two key determinants guide operative planning: tumor type and tumor location. Oncologic and radical resection remains the standard of care for malignant tumors, however, parenchyma-sparing procedures have gained interest for the treatment of benign or low-grade tumors (217). Masses and tumors located in the pancreatic head often require pancreaticoduodenectomy with or without pylorus-preservation, or duodenum-preserving pancreatic head resection (DPPHR). Total pancreatectomy may rarely be required to achieve negative surgical margins. Lesions in the pancreatic body/tail are frequently resected with a distal pancreatectomy. However, central pancreatectomy has been described in masses limited to the pancreatic neck and proximal body, and enucleation may be performed for smaller benign tumors.

Although relatively uncommon, pancreatic surgery in pediatric patients has been shown to be safe and effective, especially when performed by experienced surgeons (218–222). When feasible, laparoscopic pancreatic resections have also demonstrated similar outcomes and decreased morbidity (223, 224). In studies of pediatric patients undergoing pancreatic resection for tumors, cohorts are relatively small with heterogeneous pathology. In a study of 46 children undergoing pancreatic operations, 10 patients with SPN mostly underwent DP, followed by PD, and central pancreatectomy. Two patients with PBL underwent total pancreatectomy and PD, and three patients with desmoid tumors of the pancreas underwent DP. Four patients underwent either DP or PD for pancreatic NET resection (218). Like findings in this particular study, the literature demonstrates low surgery-related mortality rates overall (219–221).

#### Radical resection remains the gold standard for malignant disease

**Figure 12:**
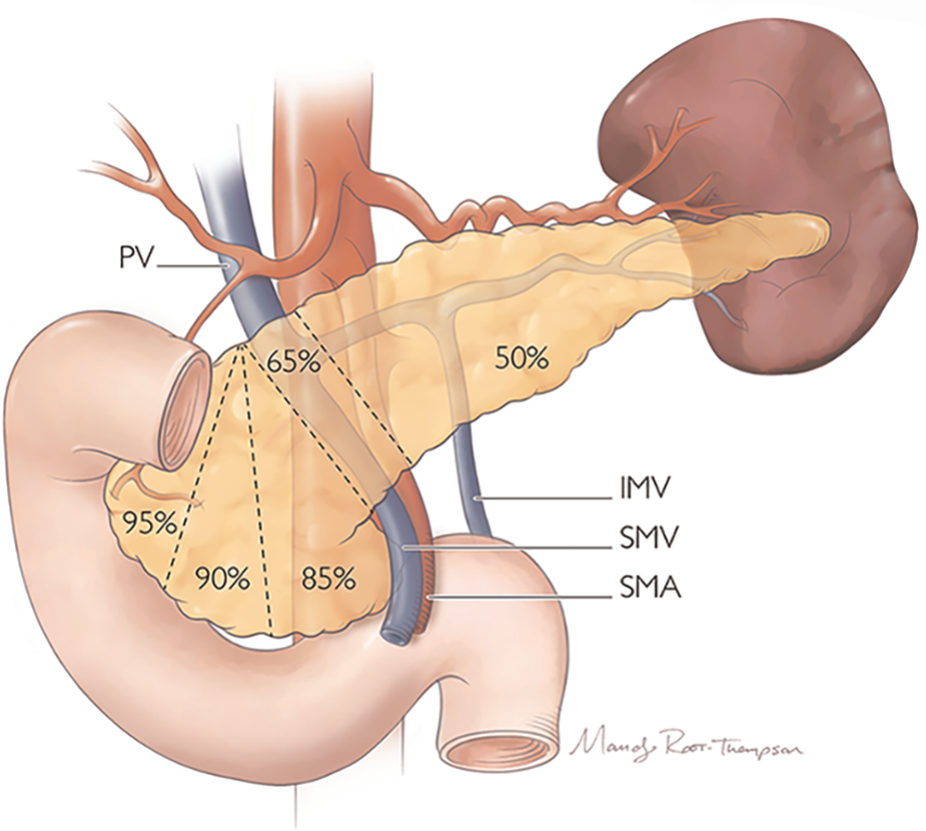
Proportions of the pancreas which may be resected during radical or parenchyma-preserving operation.

Pancreatic tumors requiring PD in children and adolescents are uncommon (219, 225). Also known as a Whipple operation, the procedure involves resection of the pancreatic head, duodenum, and distal bile duct, resulting in removal of up to 50% of the gland (Figure 12) (226). Historically, an antrectomy is performed with duodenal resection; however, the pylorus may be preserved (pylorus-preserving) by dividing the proximal duodenum and creating a duodenojejunostomy reconstruction. One of the biggest concerns after PD are the rates of endocrine and exocrine dysfunction, which may occur in up to 50% of patients (226). The removal of the duodenum, proximal jejunum, and pancreatic head eliminates important metabolic and hormonal signaling centers of the gastrointestinal tract. This leads to impairment of endocrine function in the form of new-onset diabetes mellitus (DM) and impairment of exocrine function requiring pancreatic enzyme replacement therapy (227).

In a recent meta-analysis of predominantly adults, patients undergoing PD for benign tumors developed new-onset DM in 14% of cases, while 54% developed new-onset exocrine insufficiency (228). In pylorus-preserving PD, 20% of patients developed new-onset DM, and 45% developed new-onset exocrine insufficiency. In the largest series of pediatric patients undergoing open PD, 65 children with a median age of 13 years old from 18 hospitals were evaluated (225). The most common histological diagnoses requiring PD were SPN, followed by PBL, PDAC, and pancreatic NET. Pancreatic leak occurred in 14% of patients, while 32% of patients developed pancreatic insufficiency, and 9% developed delayed gastric emptying. Overall, 22% experienced recurrence and 17% experienced mortality. In this cohort, survival and recurrence were not impacted by the type of PD, neoadjuvant chemotherapy, or the presence of an adult hepatobiliary surgeon. In a smaller study of 22 patients less than 30 years old undergoing PD, intra-abdominal abscess was the most common complication (14%), pancreatic leak occurred in 4.5%, and there were no mortalities reported (219).

DP typically involves resection of some or all of the pancreatic body/tail to the left of the superior mesenteric vein/portal vein, which results in removal of approximately 50% of the gland (226) (Figure 12). The operation may include a splenectomy, especially in cases where an oncologic resection is required, or may be spleen-preserving. Spleen-preserving DP and DP with splenectomy have similar clinical outcomes with respect to postoperative pancreatic fistula occurrence (7.6%), wound infection, and re-operation rates in patients with benign or borderline malignant tumors of the pancreas (229–232). However, the incidence of infectious complications is significantly reduced in patients who undergo spleen-preserving DP (9%) compared to those who undergo splenectomy (28%), suggesting that splenic preservation should be maximized when feasible (230). This is especially true in pediatric patients due to their increased risk for overwhelming post-splenectomy infection (OPSI) (233). Further, laparoscopic spleen-preserving DP has also been demonstrated as safe and feasible in children, specifically for treatment of SPN (233, 234).

In the meta-analysis by Beger et al., DP was associated with new-onset DM in 23% of patients and exocrine insufficiency in 17% (228). In a study of patients under 40 years old, 112 underwent DP, most commonly for pancreatic NET, mucinous cystic neoplasm, and SPN (235). However, when a subset of patients ≤18 years old were evaluated, most had pathology for SPN, followed by pancreatic NET. In patients under 40 years old, new-onset diabetes occurred in 15% and exocrine insufficiency in 16%, while 8% of patients ≤18 years old developed new-onset diabetes and none had postoperative exocrine insufficiency. Overall, there were no mortalities related to operation.

#### Parenchyma-sparing resection may be justified

A variety of surgical methods have been described with the goal to maximize the preservation of pancreatic parenchymal tissue, preserve the gastrointestinal tract anatomy and function, and avoid postoperative endocrine and exocrine dysfunction (217). Among those most commonly performed in children with pancreatic tumors are DPPHR, central pancreatectomy, and enucleation.

A DPPHR procedure was first introduced in 1972 to surgically treat inflammatory masses in the pancreatic head (236). Depending on the extent of the pancreatic head resection, DPPHR is classified as total or partial, with total DPPHR often being implemented to avoid incomplete resection in the setting of malignancy. Concurrent segmental resection of the duodenum may also be performed to obtain an appropriate oncologic resection (217). In 1994, Nako et al. described DPPHR with segmental resection of the periampullary duodenum, and in 1999, Beger reported DPHHR without segmental resection of the duodenum (237, 238). The Berne procedure was subsequently introduced as a technical simplification of the Beger procedure, as it avoids division of the pancreatic neck over the portal vein (239). Frey also reported an operation which combines DPPHR and longitudinal pancreaticojejunostomy (240). DPPHR is best suited for benign and low-grade malignant tumors in the pancreatic head including SPN, pancreatic NET, serous cystic adenoma, and intraductal papillary mucinous neoplasm (IPMN), producing favorable outcomes in these patients (217, 241). Laparoscopic DPPHR (LDPPHR) has also been proven a safe and effective surgical procedure (217, 236, 241). Compared to laparoscopic PD, LDPPHR has similar outcomes related to postoperative complications, pancreatic fistula, 30-day readmission, and 90-day mortality, while also being a shorter operation (217).

Quantitative comparisons with PD have also demonstrated both short-term and long-term benefits (242). DPPHR demonstrated less new-onset DM (5%) compared to PD (15.7%) and less exocrine insufficiency (6.7% vs. 44.3%) (228). Following PD, significant impairment was measured in the gastrointestinal hormones gastrin, motilin, insulin, secretin, PP, and gastric inhibitory polypeptide (GIP), while no change in the response of these hormones was seen after DPPHR (227). In 21 children with an average age of 11.7 years who underwent DPPHR, most had pathology for SPN (*n* = 10) (242). Thirty-three percent required exocrine enzymatic replacement therapy, though there were no mortalities. In appropriately selected patients with pancreatic head masses, DPPHR may be preferable to preserve native gastrointestinal anatomy and avoid long-term pancreatic insufficiency with its related consequences.

**Figure 13:**
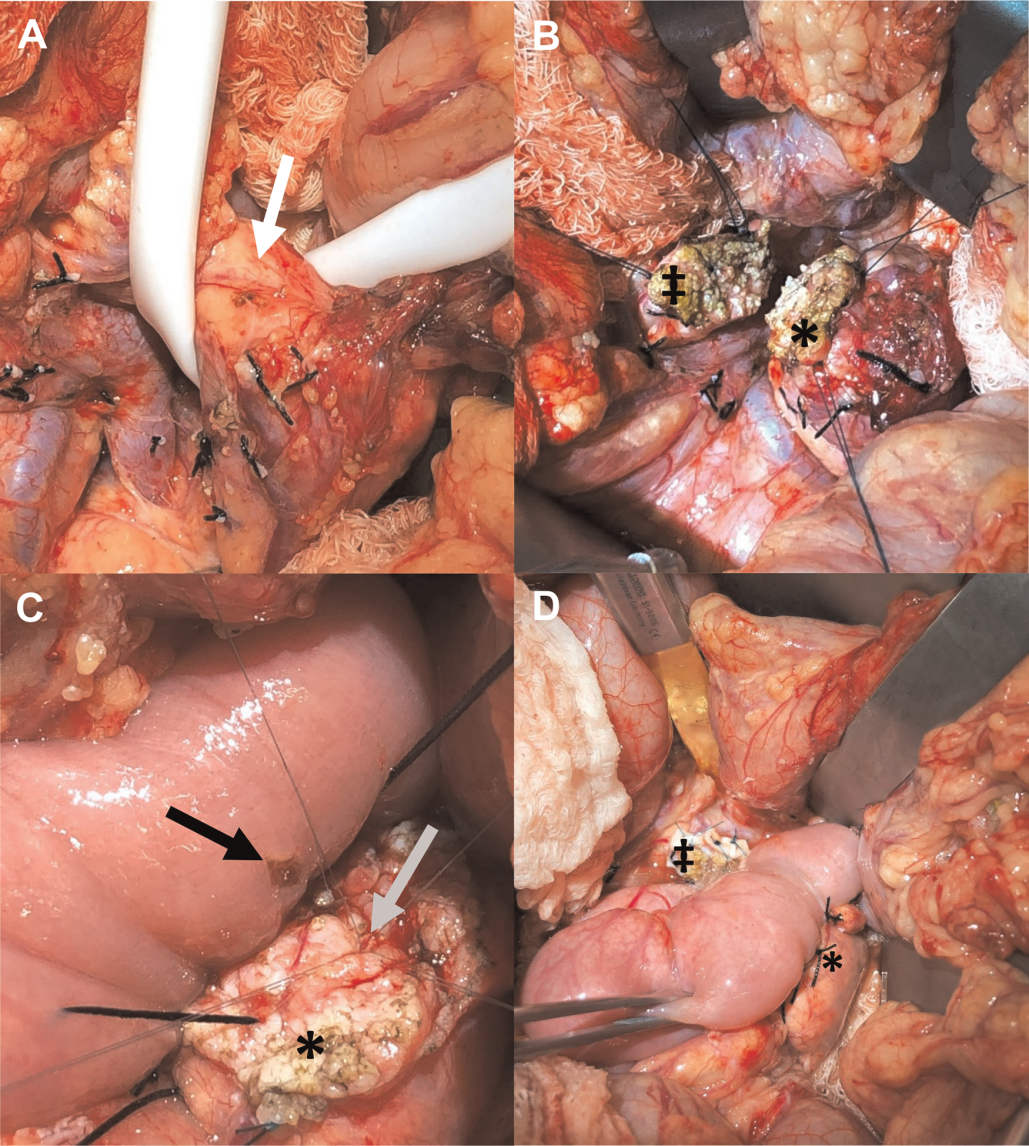
Central pancreatectomy. (**A**) Ewing sarcoma is demonstrated within the neck of the pancreatic parenchyma (white arrow). (**B**) Following resection of the neck of pancreas with mass, edges of the transected pancreatic head (‡) and body (*) are demonstrated. (**C**) For reconstruction, a Roux-en-Y jejunal enterotomy is made (black arrow), and the main pancreatic duct (gray arrow) within the distal pancreatic remnant (*) is prepared for anastomosis. (**D**) Reconstruction involving anastomosis of the Roux-en-Y jejunal limb to the distal pancreatic remnant (*) creating a complete pancreaticojejunal anastomosis. The remnant pancreatic head is also demonstrated (‡). IMV: inferior mesenteric vein; PV: portal vein; SMA: superior mesenteric artery; SMV, superior mesenteric vein.

Since central pancreatectomy was first reported in 1984 by Dagradi and Serio, at least 1,305 cases have been reported in the literature (243, 244). Often, tumors located in the pancreatic neck or proximal body create a challenge for surgeons, requiring either an extended PD or extended distal pancreatectomy. However, in benign and borderline disease, this substantial loss of normal pancreatic tissue results in increased and unnecessary endocrine and exocrine dysfunction for the patient (245). Also known as a middle or medial pancreatectomy, central pancreatectomy is a parenchyma-sparing procedure used to resect benign and low-grade malignant tumors located in the pancreatic neck and proximal body. Typically, these include SPNs, pancreatic NETs, and small tumors which are deeply embedded within the parenchyma and not amenable to enucleation (246). Typically, a Roux-en-Y jejunal limb is created, with a pancreaticojejunostomy anastomosis performed at the distal remnant pancreatic stump (Figure 13). In a meta-analysis by Iacono et al., most distal pancreatic stumps in central pancreatectomies were managed by pancreaticojejunostomy (58%) or pancreaticogastrostomy (38%), while the proximal stump was closed by suturing (64%) or stapling (30%) or anastomosed via pancreaticojejunostomy (6%).

Mortality rates following central pancreatectomy in adults are low (0.5%–0.8%), and pancreatic fistula is the most common complication, reported in 35%–41% of cases (243, 246). However, the use of a pancreaticogastrostomy anastomosis has been associated with significantly higher pancreatic fistula incidence and severity compared to pancreaticojejunostomy (247). Pancreatic fistula rates following central pancreatectomy are also higher compared to rates after PD or DP (246). Compared to PD, central pancreatectomy is associated with lower intraoperative blood loss, shorter operative time, and shorter hospital stay. However, compared to DP, central pancreatectomy is associated with longer operative time and hospital stay. New-onset DM and exocrine insufficiency is significantly lower following central pancreatectomy, compared to both PD and DP (246).

Evidence related to central pancreatectomy performed in children is limited to case series and reports but has been reported for the management of PBL and SPN, where complete surgical resection was possible (218, 248–251). Two cases are reported with the use of a pancreaticogastrostomy anastomosis, and two other cases are reported using a Roux-en-Y jejunal limb, with one being performed robotically (248, 249). One 16-year-old with PBL underwent resection and adjuvant chemotherapy, which led to prolonged disease-free survival without the development of pancreatic endocrine and exocrine insufficiency (250). Central pancreatectomy has also been reported for resection of a Ewing sarcoma in a young child who had undergone neoadjuvant chemotherapy (Figure 13) (8). Overall, although pancreatic fistula occurrence is higher than that in PD and DP, central pancreatectomy provides important clinical benefits due its ability to preserve normal pancreatic parenchyma in pediatric patients with expected long-term survival.

Enucleation has also been considered reasonable surgical management for benign and low-grade malignancies. However, it should be avoided in certain tumors greater than 2 cm in size (i.e., non-functioning pancreatic NET), embedded deep within the gland, located less than 2–3 mm from the main pancreatic duct, and without clearly benign pathology (i.e., margins cannot be compromised) (252–254). Enucleation is most commonly performed for insulinomas and non-functioning pancreatic NETs. It is particularly useful for benign symptomatic pancreatic NETs, like insulinoma, because it provides excellent outcomes even when margins are positive, while also maintaining quality of life for the patient. Varying rates of pancreatic fistula have been reported (21%–61%). Although these rates are sometimes higher than that seen in standard resection, they do not necessarily result in higher morbidity and mortality (254, 255). In a large series, new-onset DM was demonstrated in 0%–5% of patients undergoing enucleation, while exocrine insufficiency has been reported in just 4% (228, 254).

## Conclusion

In conclusion, pancreatic tumors are uncommon in children but have better survival and overall outcomes compared to adults with pancreatic tumors. PBL and SPN are the most common pancreatic tumors diagnosed in children and are best managed by complete surgical resection. Insulinomas remain the most common pancreatic NET and may undergo enucleation in certain cases where tumors are small (<2 cm) and benign. Although some tumors overlap in their clinical presentation and imaging characteristics, AIP may be especially challenging to distinguish from PDAC, resulting in unnecessary radical pancreatic resections. However, EUS has become an important adjunct in diagnostic imaging and may provide helpful guidance toward an accurate diagnosis. Lastly, while malignant tumors require radical oncologic resection, often with PD or DP, parenchyma-sparing surgical management should be a considered alternative for benign and low-grade malignancy, as it has been shown to be safe and effective while preserving pancreatic endocrine and exocrine function in children.
